# Successful Treatment of Delayed Partial Flap Loss in Autologous Breast Reconstruction With Rivaroxaban

**DOI:** 10.7759/cureus.95094

**Published:** 2025-10-21

**Authors:** Jadon H Beck, Anne M Meyer, Kerilyn Godbe, James A Butterworth, Eric C Lai

**Affiliations:** 1 School of Medicine, University of Kansas, Kansas City, USA; 2 Plastic Surgery, University of Kansas Medical Center, Kansas City, USA

**Keywords:** autologous breast reconstruction, breast plastic surgery, delayed partial flap loss, microvascular venous congestion, plastic and reconstructive surgery

## Abstract

Modern free flap breast reconstruction has high levels of success and patient satisfaction. When delayed microvascular complications do arise, corresponding salvage rates are low. Here, we present the case of a 72-year-old female who underwent uneventful right autologous breast reconstruction using profunda artery perforator (PAP) stacked flaps, whose course was complicated at postoperative day eight by partial superior flap congestion. Successful salvage of the superior PAP flap was achieved after three weeks of rivaroxaban treatment. To our knowledge, this case is the first in the literature to suggest the potential benefit of systemic anticoagulation in treating delayed partial flap congestion.

## Introduction

Free flaps are generally a very successful option for breast reconstruction, with low rates of complications. Free flaps have been shown to be associated with increased patient satisfaction due to the more natural appearance of the breast compared to implant-based reconstruction and avoid complications of implant reconstruction such as capsular contracture and implant rupture [1-3]. Free flap autologous breast reconstruction involves taking tissue from the patient, in this case, the upper posterior thigh, to the chest to reconstruct a breast mound. Blood supply to the flaps is then established by microvascular anastomosis between the profunda artery perforators (PAPs) and the accompanying vein to the internal mammary vascular. Microvascular complications are a common complication, with high rates of salvage when detected promptly [1,4]. A delayed presentation of flap compromise can be an infrequent but devastating complication, resulting in challenges with salvage and overall management [3,5].

Surgical exploration remains the gold standard for flap salvage in the immediate postoperative period. Treatment options for delayed flap loss are less standardized, including catheter-directed thrombolysis, medicinal leeches, and hyperbaric oxygen therapy [6-11]. Due to the lack of effectiveness and safety in these options, standard treatment has yet to be identified.

## Case presentation

**Figure 1:**
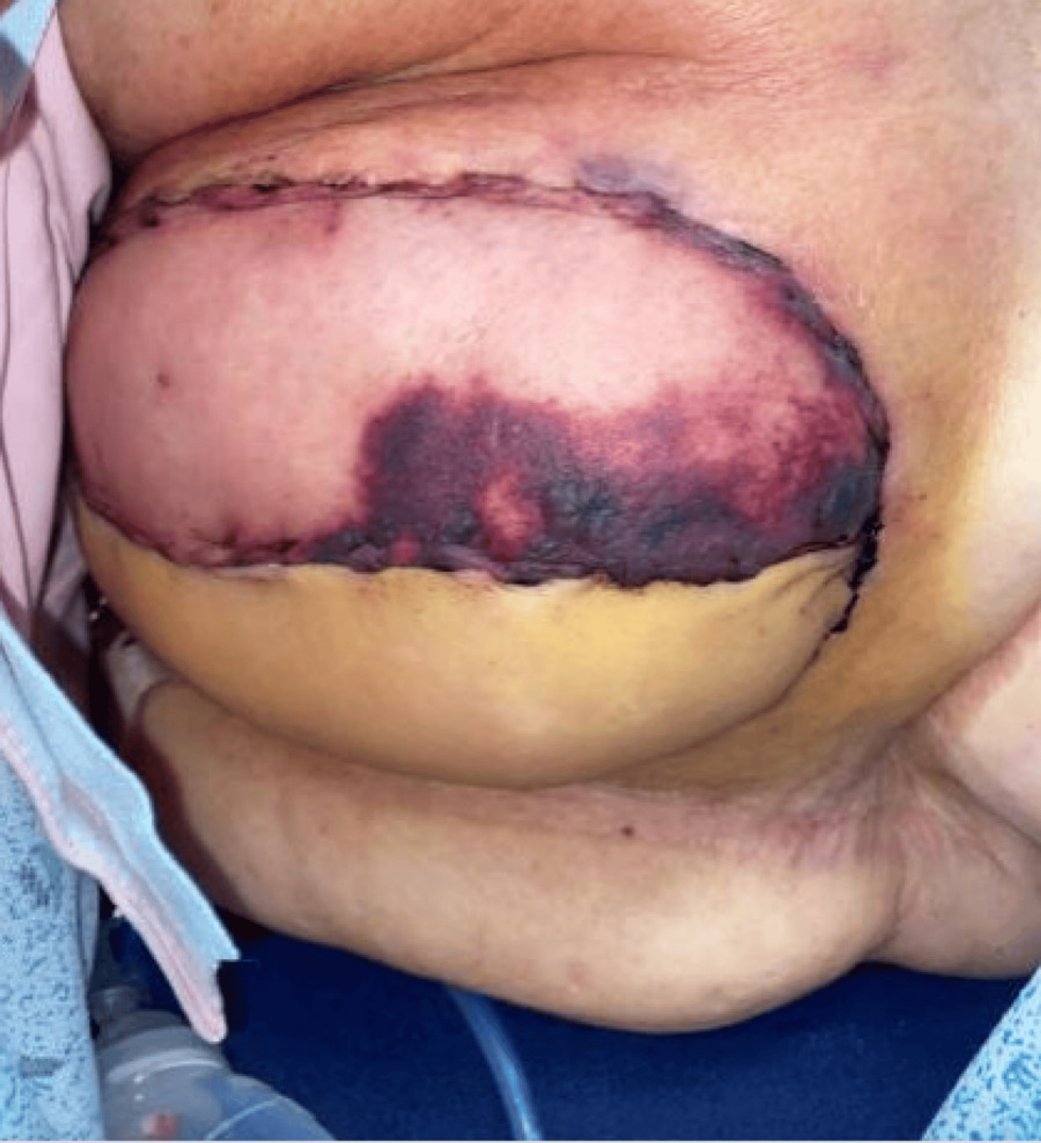
Presentation of the patient’s right breast on day eight postoperatively demonstrating skin changes and edema of the right superior profunda artery perforator flap.

A healthy 72-year-old Caucasian female was diagnosed with right grade II invasive ductal carcinoma of the breast, estrogen receptor positive and negative for progesterone and HER2 receptor, in 2022. Following a one-month course of radiology with a total radiation dose of 5,005 cGy, the patient elected to proceed with a right skin-sparing mastectomy with placement of a tissue expander and acellular dermal matrix. One and a half years following mastectomy, she underwent right breast reconstruction with stacked PAP free flaps. The superior breast flap was constructed using a right lower extremity PAP flap with one perforator, and the inferior breast flap was constructed using a left lower extremity PAP flap with two perforators. Microvascular anastomoses were performed, joining the internal mammary vessels to the profunda vessels. The superior breast flap was joined with a 2.5 mm venous coupler to the retrograde limb of the internal mammary vein, and the inferior breast flap was anastomosed with a 2.0 mm venous coupler to the anterograde limb of the internal mammary vessel. There were no concerns regarding flap viability during the operation. The immediate inpatient recovery was uneventful, and the patient was discharged postoperatively on day three with no evidence of skin changes, discoloration, or fluid collections and biphasic arterial and audible venous Doppler signals. At her first postoperative visit on day eight, the superior PAP flap demonstrated skin changes and pitting edema but no palpable fluid collection (Figure 1). The flap had a strong arterial Doppler signal. The patient was unable to provide a timeline for the noted changes. The delayed presentation and examination were most concerning due to presumed venous congestion and partial impending flap loss. She was admitted for possible operative exploration versus conservative management. Further imaging was not pursued given the lack of palpable fluid collection on examination. The decision was made to manage the partial flap loss and presumed venous congestion with local wound care, including daily bacitracin, and systemic anticoagulation on an outpatient basis, as it was believed the patient would not benefit from further exploration, and exploration might risk full compromise. Additionally, the patient was not interested in further surgical interventions at this time. She was prescribed 10 mg of rivaroxaban daily for a total of three weeks at discharge. The prophylactic dosage of 10 mg was chosen due to concern for the development of a hematoma at therapeutic dosage. Rivaroxaban was chosen due to the ease of use and the routine monitoring of international normalization ratio and coagulation studies being unnecessary.

**Figure 2:**
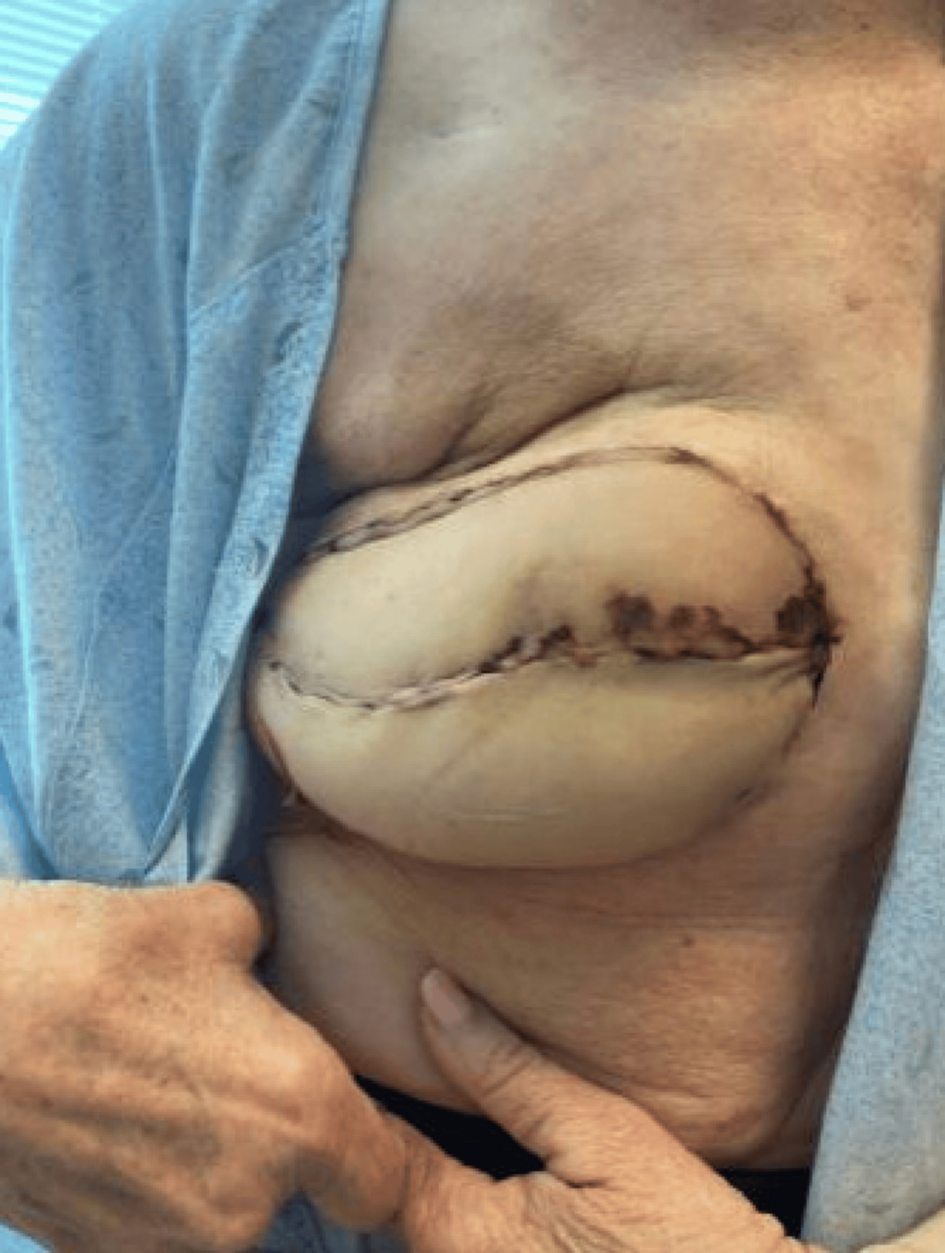
Patient’s right breast on day 22 postoperatively with significant improvement of superior profunda artery perforator flap color and edema.

After two weeks of rivaroxaban and local wound care, the appearance of the flap skin improved significantly (Figure 2). There was continued improvement at two months postoperatively, with complete resolution at four months. The patient experienced no adverse side effects of rivaroxaban treatment. Aesthetically, hollowing of the superior pole of the right breast was present, for which the patient elected to proceed with fat grafting to improve contour and symmetry and concomitant nipple reconstruction.

## Discussion

Microvascular compromise is a serious complication of autologous breast reconstruction [3-5]. The highest risk occurs intraoperatively and within the first 48 hours postoperatively, as the flap depends entirely on the pedicle for perfusion [4,5,12]. Beyond this critical period, the causes of microvascular compromise are more varied and less well understood, including infection, mechanical obstruction from edema, and issues with flap inset. The benefits of re-exploration with resultant salvage after the immediate postoperative period have not been well supported in the literature, with some even reporting that operative salvage is not feasible [5,12,13]. This case presents the intriguing use of systemic anticoagulation in cases of presumed venous thrombosis following delayed partial flap compromise.

Given the delayed presentation, slow progression, and a strong arterial Doppler signal, a technical issue with the primary anastomosis was unlikely, thereby limiting the potential benefit of surgical exploration. The dusky appearance and edema of the right superior PAP flap suggested venous congestion, possibly due to microthrombi formation. The smaller venous vessels, with their thin walls, are particularly vulnerable to compression and collapse, creating a prothrombotic environment [12,14].

Managing delayed partial flap loss varies depending on the surgeon’s preference and patient factors. Surgical exploration remains the gold standard when microvascular compromise is suspected in free flaps in the immediate postoperative phase [1,5]. However, surgical intervention may not be beneficial in cases of delayed presentation due to the heightened inflammatory state, which increases the risk of pedicle injury as well as disrupting the neovascularization, allowing the flap to remain viable [2,15].

Catheter-directed thrombolysis with thrombolytics offers a less invasive and more targeted approach than surgical exploration, but it requires an additional procedure and carries the risk of systemic thrombolytic dissemination [9,10]. Leech therapy is another option for managing venous congestion in free flaps. However, in this case, the slow presentation of venous congestion in the superior PAP flap and challenges such as limited availability, risk of infection from leech gut bacteria, and the potential need for blood transfusion due to blood loss made this approach suboptimal [6,7].

Hyperbaric oxygen therapy (HBOT) is a minimally invasive option for salvaging free flaps. However, HBOT requires burdensome protocols, with a significant investment of time and substantial disruption to a patient’s daily life, to be successful (90-minute sessions five times per week for approximately six weeks, posing a significant logistical burden that is not feasible for many patients) [8].

This report presents the first documented case suggesting the potential benefit of systemic anticoagulation in managing delayed partial flap loss. It highlights the successful use of a three-week course of systemic anticoagulation with rivaroxaban to manage delayed microvascular compromise. This allows the patient to avoid a longer inpatient stay with the use of leeches, as suggested by some, and avoid an operation that would likely not improve the salvage rate [2,15]. Rivaroxaban is a fast-acting, direct factor Xa inhibitor that prevents the conversion of prothrombin to thrombin, offering high oral bioavailability. Previous studies have demonstrated its effectiveness in preventing further propagation of venous thrombi when administered after thrombus formation [16].

Animal studies suggest that rivaroxaban downregulates the TLR4/NF-kB/NLRP3 signaling pathway, reducing inflammation and improving flap survival. As this pathway is implicated in tissue necrosis and ischemia-reperfusion injury, its inhibition may have contributed to the flap’s successful salvage [17].

However, the risks of rivaroxaban must be carefully evaluated before initiation. It is contraindicated in patients with renal impairment and those receiving systemic treatment with CYP3 inhibitors, P-glycoprotein inhibitors, or HIV protease inhibitors [16]. In this case, the patient was otherwise healthy, and after a thorough discussion of the risks and benefits, she opted to proceed with treatment.

This case represents our experience with a single patient, with an unusual presentation and limited literature for guidance regarding management. We are not proposing this treatment as a standard of care; however, if other providers find themselves presented with a similar challenging case, we hope this data point may be helpful.

## Conclusions

Delayed partial flap loss is a challenging complication to manage following free flap reconstruction procedures. Here, we demonstrate the successful use of systemic anticoagulation to address this issue in this patient. This treatment is not generally applicable and may not be suitable for patients with varying risk profiles. Further studies are necessary before making direct associations between rivaroxaban and flap salvage.
